# Stevens-Johnson Syndrome/Toxic Epidermal Necrolysis Should Be Kept in Mind in Children with Febrile Neutropenia, Oral Cavity Lesions, and Skin Rash

**DOI:** 10.4274/tjh.2014.0470

**Published:** 2016-05-16

**Authors:** Eda Ataseven, Şebnem Yılmaz Bengoa, Hale Ören

**Affiliations:** 1 Dokuz Eylül University Faculty of Medicine, Department of Pediatric Hematology, İzmir, Turkey

**Keywords:** Acute leukemia, Stevens-Johnson syndrome, Toxic epidermal necrolysis

A 14-year-old boy was diagnosed with acute lymphoblastic leukemia. Febrile neutropenia developed during induction. Imipenem and teicoplanin were started because of severe mucositis. Viral tests and bacterial cultures were unrevealing. On follow-up, a painful papular rash had appeared and oral mucositis had become worse (Figure 1). Stevens-Johnson syndrome (SJS)/toxic epidermal necrolysis (TEN) was suspected. Intravenous immunoglobulin (IVIG) at 1 g/kg/day and methylprednisolone at 1 mg/kg/day were started. The lesions regressed in 1 week (Figure 2). Skin biopsy was consistent with SJS/TEN. Informed consent was obtained.

SJS and TEN are rare diseases characterized by fever and mucosal and cutaneous lesions [1]. It is defined as SJS when epidermal involvement affects less than 10% of the body surface area, as SJS/TEN overlap when the skin detachment ranges from 10% to 30%, and as TEN when it involves more than 30% [1,2]. It may occur after taking a new medication or may rarely have an infectious origin. Our patient had no predisposing conditions other than taking chemotherapeutic drugs and antibiotics. The mortality rate is high in SJS/TEN [1,3]. Rapid withdrawal of the probable causative drug(s) is important. Use of IVIGs and corticosteroids is reported as the most commonly used therapy in childhood [1,4]. Systematic review of adult treatments for SJS and TEN did not show any benefit of these agents on mortality rates [3]. Cyclosporine, plasmapheresis, and tumor necrosis factor-alpha inhibitors have been also reported among other treatment options [1,2,3,4,5].

## Figures and Tables

**Figure 1:**
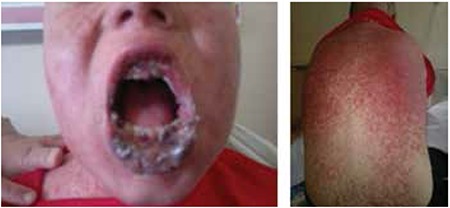
Erosions and crusts on the lips and hemorrhagic ulcers in the oral cavity. A red papular rash spread to the shoulders and the back.

**Figure 2:**
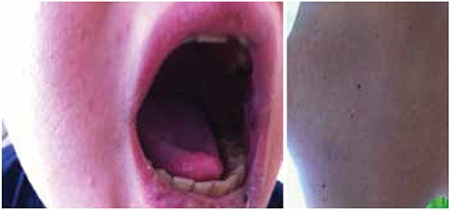
Regression of the lesions on the lips, oral cavity, and back.
